# Game of microbes: the battle within – gut microbiota and multiple sclerosis

**DOI:** 10.1080/19490976.2024.2387794

**Published:** 2024-08-08

**Authors:** Ti-Ara Turner, Peter Lehman, Sudeep Ghimire, Shailesh K. Shahi, Ashutosh Mangalam

**Affiliations:** a Interdisciplinary Graduate Program in Immunology, University of Iowa, Iowa City, IA, USA; b Iowa City VA Health Care System, Iowa City, IA, USA; c Experimental Pathology Graduate Program, University of Iowa, Iowa City, IA, USA; d Department of Pathology, Carver College of Medicine, University of Iowa, Iowa City, IA, USA

**Keywords:** Multiple sclerosis, gut microbiome, diet, fiber, inflammation, therapeutic, phytoestrogen, short-chain fatty acids, tryptophan, LPS

## Abstract

Multiple sclerosis (MS) is a chronic and progressive autoimmune disease of the central nervous system (CNS), with both genetic and environmental factors contributing to the pathobiology of the disease. While human leukocyte antigen (HLA) genes have emerged as the strongest genetic factor, consensus on environmental risk factors are lacking. Recently, trillions of microbes residing in our gut (microbiome) have emerged as a potential environmental factor linked with the pathobiology of MS as PwMS show gut microbial dysbiosis (altered gut microbiome). Thus, there has been a strong emphasis on understanding the factors (host and environmental) regulating the composition of the gut microbiota and the mechanism(s) through which gut microbes contribute to MS disease, especially through immune system modulation. A better understanding of these interactions will help harness the enormous potential of the gut microbiota as a therapeutic approach to treating MS.

## 1. Pathobiology of multiple sclerosis

Multiple sclerosis (MS), a chronic autoimmune disorder of the central nervous system (CNS) affecting an estimated 2.8 million individuals globally, is a significant contributor to neurological disability in young adults.^1,2^ MS is categorized into various subtypes based on radiological and pathological findings, each with distinct clinical courses and prognoses.^3^ The most common form, relapsing-remitting MS (RRMS), is characterized by episodes of neurological symptoms followed by periods of remission. The progressive forms of MS include primary progressive MS (PPMS) and secondary progressive MS (SPMS), where disability steadily accumulates over time.^3^ Additionally, radiologically isolated syndrome (RIS) and clinically isolated syndrome (CIS) are recognized as pre-MS stages, with RIS characterized by radiological evidence of demyelination without clinical symptoms and CIS presenting with a first clinical episode suggestive of demyelination but not yet fulfilled the criteria for a definitive MS diagnosis.^3^

MS pathogenesis results from multifactorial etiology involving genetic and environmental factors.^4,5^ The Human Leukocyte Antigen (HLA) gene complex, which plays a critical role in the immune system, has emerged as a critical genetic risk factor in MS, with certain HLA-class II genes having been linked with an increased risk of developing MS.^6,7^ In addition to the HLA gene, other genetic variants have also been implicated in MS susceptibility, including genes involved in immune regulation, myelin formation, and neuronal signaling.^8^ However, studies of monozygotic twins have shown that genetic factors contribute only ~30% of the disease risk,^9^ with the rest linked to environmental factors. Environmental factors, such as gut microbiota, vitamin D deficiency, smoking, diet, and infections such as Epstein–Barr virus, have been linked with MS.^5^ In the last decade, gut microbiota dysbiosis, an imbalance in the composition and function of gut bacteria, has emerged as a potential environmental factor contributing to the pathobiology of MS.^10^

The underlying pathophysiology of MS involves a dysregulated immune response, primarily driven by the infiltration of T cells, B cells, and macrophages into the central nervous system (CNS). Activated T cells, particularly T-helper 1 (Th1) and T-helper 17 (Th17) subsets, secrete pro-inflammatory cytokines such as interferon (IFN)-γ, tumor necrosis factor (TNF)-α, Granulocyte-Macrophage Colony-Stimulating Factor (GM-CSF), and IL-17, leading to inflammation, demyelination, and axonal damage.^11,12^ While the initial triggers of MS remain elusive, a prevailing hypothesis suggests that in genetically predisposed individuals, autoreactive CD4+ T cells targeting central nervous system (CNS) myelin antigens become activated in the periphery.^11,12^ These activated CD4+ T cells, secreting pro-inflammatory cytokines, breach the blood–brain barrier, a process facilitated by the interaction of alpha 4 beta 1 integrins on their surface with Vascular Cell Adhesion Molecule VCAM-1 expressed on endothelial cells.^13^ This critical interaction allows for the transmigration of these autoreactive T cells into the CNS, where they initiate an immune assault on the myelin sheath-insulating neuronal axons. This dysregulated immune response triggers a cascade of inflammation, recruiting additional immune cells such as B cells, macrophages, and neutrophils from the periphery and activating glial cells into pro-inflammatory phenotypes, culminating in demyelination and axonal damage.^14^ This pathological process manifests in a wide array of debilitating clinical symptoms, including optic neuritis, muscle weakness, sensory disturbances, fatigue, and cognitive impairment.

Under normal physiological conditions, robust immune regulatory mechanisms prevent inflammatory cascades that can lead to inflammation and demyelination in MS. Typically, autoreactive CD4+ T cells are held in check by immunoregulatory cells, such as regulatory CD4+ T cells expressing FoxP3 and/or IL-10 producing type 1 regulatory cells (Tr1).^15^ Additionally, other immune cells, such as CD8+ T cells, B cells, and NKT cells, can regulate autoreactive immune responses.^15,16^ However, in genetically predisposed individuals, environmental triggers can overcome these tolerance mechanisms, enabling autoreactive T cells to initiate pathogenic processes. The importance of CD4 and CD8 T cells in the pathobiology of MS is validated by a defective immunoregulatory response characterized by reduced number and/or function of regulatory immune cells during disease relapse.^15,16^

The gut microbiome communicates with the brain through various pathways, including neural and immune signaling. This bidirectional communication, known as the gut-brain axis, highlights the potential for gut bacteria and their metabolites to influence pathobiology of MS. Given the association between dysregulated immune response and MS, gut dysbiosis, which can influence immune regulation, may contribute to the development and exacerbation of MS. Disruptions in the gut microbial community can lead to increased intestinal permeability, allowing bacterial products and toxins to leak into the bloodstream, triggering the production of pro-inflammatory cytokines, and potentially facilitating immune cell infiltration into the CNS.^17^ Additionally, certain gut bacteria may directly modulate immune responses, promoting the development of autoreactive pro-inflammatory T cell subsets.^18^ Finally, gut bacteria produce a variety of metabolites such as short-chain fatty acids (SCFAs), tryptophan metabolites, phytoestrogen metabolites, and bile acids that can directly or indirectly impact the nervous system.^19–22^

This review will focus on how gut microbiota and their metabolites might contribute to the pathobiology of MS through the modulation of inflammatory responses in the periphery and CNS. Deciphering the intricate interplay between the pro-inflammatory immune response and the gut microbiome is paramount to a comprehensive understanding of MS pathobiology. This knowledge holds immense potential for harnessing gut microbiota manipulation as a therapeutic strategy to mitigate the debilitating effects of MS. Modulating the gut microbiome and dietary interventions may offer novel avenues for immunomodulation and disease management, ultimately improving the quality of life for individuals living with MS.

## 2. Gut microbiota and human health

The human gastrointestinal tract is colonized by trillions of diverse microorganisms, such as viruses, bacteria, and fungi, collectively called the gut microbiome.^23^ These highly diverse communities have the potential to influence human health systemically. The gut microbiome forms a symbiotic relationship with the host, where the host provides food and space for microbes to survive and grow while the microbes aid in maintaining the host’s health by helping with various physiological processes.^24,25^ A diverse gut microbiome develops in humans from birth until approximately 3 years of age; however, the specific microbes present can be altered by host-derived and environmental factors.^26^ Examples of host factors include molecules produced by intestinal epithelial cells, including mucus, antimicrobial peptides, immunoglobulin A (IgA), and MicroRNAs (miRNAs). Environmental factors like diet, lifestyle, medications, age, and delivery pattern can alter the gut microbiome’s composition.^27^ Changes in gut microbiota composition can perturb a balanced ecology characterized by reduced microbial richness, loss of beneficial microbes, and increases in pathobionts – commonly known as dysbiosis.^28^ This review will discuss the role of gut microbiota and diet as environmental factors in the pathobiology of MS.

The gut microbiome can affect various functions throughout the human body. Recent studies have noted that the gut microbiome can maintain bidirectional communication with the CNS.^29,30^ Through the vagus nerve, neuroendocrine system (ENS), and immune system, the CNS can directly or indirectly influence gut functions, such as nutrient uptake, gut permeability, and mucus production.^31^ On the other hand, the gut microbiome can communicate with the CNS through metabolite production that can act on the CNS directly or indirectly by activating immune cells that interact with it.^21,22,32^ Moreover, PwMS are reported to have symptoms such as constipation, diarrhea, and gastrointestinal (GI) discomfort.^33–35^ Although dysbiosis can be detected in these patients, there is no specific gut microbiome compositional signature for individual diseases, thus emphasizing the importance of better understanding how the gut microbiome may be linked to neurodegenerative diseases like MS.

## 3. Links between the gut microbiome and MS

Several studies, including from our groups, have shown that people with MS (PwMS) have gut dysbiosis characterized by a distinct gut microbiome compared to sex- and age-matched healthy controls.^36–48^ Specifically, PwMS show enrichment of gut bacteria such as *Ruminococcus, Blautia, Dorea, Bifidobacterium, Bilophila, Sutterella, and Akkermansia* (Table 1). Conversely, the genera *Clostridium, Faecalibacterium, Eubacterium, Ruminococcus, Butyricimonas, Bacteroides*, and *Prevotella* showed reduced abundance in PwMS (Table 1). These studies are further supported by the mouse model of MS, known as experimental autoimmune encephalomyelitis (EAE), of which the transplantation of MS patient gut bacteria to germ-free mice resulted in exacerbated EAE disease compared to healthy controls.^36,38^ Additionally, PwMS have also been shown to have distinct mycobiome (fungus).^46,49^

**Table 1.** Summary of MS microbiome studies.

MS type and sample size	Lower abundance in PwMS	Increased abundance in PwMS	Geographical location (Reference)
RRMS (*n* = 20) HC (*n* = 40)	*Bacteroides* (*B*. *stercoris*, *B*. *coprocola*, *B*. *coprophilus)* *Fecalibacterium sp*. *Prevotella* (*P. copri*) *Anaerostipes sp*. *Clostridium sp*. *Sutterella* (*S. wadsworthensis)*	*Bifidobacterium sp*. *Streptococcus sp*. *Streptococcus thermophilus* E*ggerthella lenta*	Japan 43
RRMS (*n* = 31) HC (*n* = 36)	*Prevotella sp*. *Parabacteroides sp*. *Adlercreutzia sp*. *Collinsella sp. sp*. *Lactobacillus*	*Pedobacter sp*. *Pseudomonas sp*. *Mycoplasma sp*. *Haemophilus sp*. *Blautia sp*. *Dorea sp.*	USA 39
RRMS (*n* = 60) HC (*n* = 43)	*Butyricimona sp*. *Prevotella sp*. *Parabacteroides sp.*	*Methanobrevibacter sp*. *Akkermansia sp.*	USA 42
RRMS (*n* = 30) HC (*n* = 14)	*Eubacterium eligens* *Prevotella copri* uncultured *Bacteroides* sp. uncultured *alpha Proteobacterium* uncultured *Pseudomonas* sp	*Faecalibacterium* sp. *Ruminococcus* sp. uncultured *Oscillospiraceae sp*. uncultured *Blautia* sp. *Anaerostipes* sp. *Clostridium bolteae* uncultured *Dialister* sp. *Alistipes onderdonkii* *Bifidobacterium longum* *Coriobacterium* sp	UK 48
RRMS (*n* = 71) HC (*n* = 71)	*Parabacteroides distasonis*	*Akkermansia muciniphila* *Acinetobacter calcoaceticus*	USA 38
RRMS (*n* = 24) HC (*n* = 25)	*Bifidobacterium longum* *Clostridium leptum* *Faecalibacterium prausnitzii* *Bacteroides thetaiotaomicron* unclassified *Escherichia Anaerostipes sp*. *Prevotella sp.*	unclassified *Parabacteroides*	USA 37
RRMS (*n* = 19) HC (*n* = 17)	*Prevotella sp.*	*Streptococcus sp.*	Italy 40
Monozygotic twin pairs: MS (*n* = 34) Unaffected twin (*n* = 34)	*Adlercreutzia sp.*	*Akkermansia sp.*	Germany 36
POMS (*n* = 20) HC (*n* = 20)	*Pseudomonas Corrgata* *Haemophilus Influenzae* *Kochuria* *Sphingopyxis sp.QXT-31*	*Methanobrevibacter smithi* *Moribacter* *Mycoplasma bovoculi* *Diaphorobacter polyhydrobutyrativorans* *Methanobrevibacter millerae* *Arcanobacterium sp 2701*	Canada 47
RRMS (*n* = 20) HC (*n* = 30)	*Barnesiella sp*. *Odoribacter sp*. *Oscillospiracecae UCG 003*	*Blautia Sp* *Eggerthela sp*. *Hungatella sp.*	USA 46
PwMS (*n* = 576) Paired HC (*n* = 1152)	*Faecalibacterium prausnitzii* *Blautia species*	*Akkermansia muciniphila* *Ruthenibacterium lactatiformans* *Hungatella hathewayi* *Eisenbergiella tayi*	USA, Spain, UK, and Argentina 41

Abbreviations: RRMS, Relapsing Remitting Multiple Sclerosis (MS); HC, Healthy Control; POMS, Pediatric onset MS; PwMS, People with MS.

Although cross-sectional studies have helped establish a link between gut microbiota and MS, the question remains whether gut microbiota causes disease or disease causes dysbiosis. A recent study suggests a direct correlation between species richness and the number of disease relapses.^44^ The clinically non-active (non-relapsing) patients showed enrichment of *Faecalibacterium prausnitzii*, *Gordonibacter urolithinfaciens, Anaerostipes hadrus*, *Gemmiger formicilis*, and *Roseburia inulinivorans* compared to clinically active patients (who had at least one relapse in follow-up period). In contrast, the clinically active group showed enrichment of *Methanobrevibacter smithii* and *Victivallis vadensis*. Interestingly, bacterial species enriched in clinically active treatment-naïve cases were positively associated with circulating levels of proinflammatory cytokines IL-17A, IFN-γ, and TNF-α. Thus, this study strongly suggests that gut microbiota can directly contribute to the severity of MS disease. Data from MS microbiome studies are compiled in recent reviews^10,50,51^ and Table 1. In a study of pediatric PwMS, microbial alpha and beta diversities were not associated with relapses.^52^ However, they found that *Butyricicoccus desmolans, Odoribacter splanchnic, Lacnhospiraceae NK4A136*, and *Ruminococcaceae species* were associated with lower hazard to MS relapse while *Blautia terrorism, Lachnoclostridium, Lachnospiraceae_UCG-004*, and *Coriobacteriales* were associated with higher hazard to MS relapse.^52^ Although no significant associations of metabolic pathways to relapse were observed, super-pathways of L-tyrosine and L-phenylalanine biosynthesis were associated with a lower hazard of MRI outcomes. These studies imply that the gut microbiota is altered in relapses; however, further studies are required to determine whether microbiome modulation during relapses can alter the severity of RRMS outcomes in patients.

**Figure 1.**
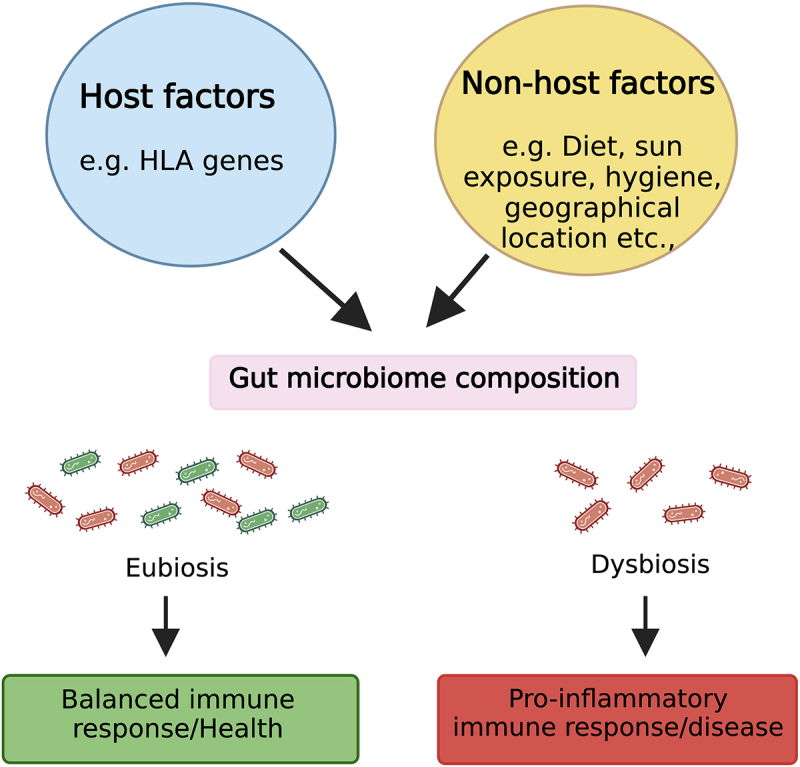
Factors affecting gut microbiota and a potential mechanism through which gut microbiota affects the host during health and disease. Host-specific factors such as host genetics and non-host factors like diet can influence gut microbiota composition. During homeostasis, an eubiotic gut microbiota maintains a diverse beneficial microbiota (symbiont) that induces a balanced immune response. However, during dysbiosis, there is depletion and/or enrichment of pro-inflammatory microbiota (pathobionts), which shift the balance between pro and anti-inflammatory responses toward an inflammatory phenotype linked with multiple diseases, including MS. Figure created with BioRender.com.

The altered gut microbiome observed in MS has fueled intense research interest in elucidating the factors shaping this microbial community and the mechanisms by which these microbes might influence MS pathogenesis (Figure 1).

## 4. Factors affecting composition and function of gut microbiota

### 4.1. Host genetic factors, gut microbiota, and multiple sclerosis

Host genetics strongly influence gut microbiome composition, highlighted by twin studies where the gut microbiome of monozygotic twins showed more similarity than dizygotic twins’ microbiome.^9,53,54^ The importance of host genes on the microbiome was further validated by large genome-wide association studies (mGWAS), which cataloged the association between host gene variants and the gut microbiome.^55,56^ Among all the genetic factors linked with MS, major histocompatibility complex (MHC) or HLA genes show the strongest association with MS susceptibility. The HLA class-II linkage in MS differs in various populations, but the highest association is with HLA-DR2 (DRB1 × 1501)/DQ6 (DQB1 × 0602),^57^ followed by DR3/DQ2 and DR4/DQ8 haplotypes.^6,55,58^ A study involving 3,002 public human gut microbiota datasets showed that individuals with functionally similar HLA haplotypes are also similar in the microbiota composition,^59^ suggesting a possible linkage between genetics and the microbiome in MS predisposition.

However, there are limited data on the role of HLA class-II restricted gut microbiota in the modulation of disease in MS/EAE. A direct role of HLA class-II genes on gut microbiota was suggested in Myelin Basic Protein (MBP)-specific T-Cell Receptor (TCR) transgenic mice on HLA-DRβ1 × 1501 background, which developed spontaneous EAE.^60^ Utilizing HLA class-II transgenic mice, we have shown that HLA class-II polymorphism modulates gut microbiota composition, which may be responsible for the difference in disease phenotype between single and double HLA-II transgenic mice in EAE.^61,62^ Specifically, while HLA-DQ8 mice are resistant to EAE, HLA-DR3.DQ8 mice develop more severe disease than HLA-DR3 mice in an IL-17A-dependent manner. Prior studies have shown that certain gut bacteria can induce IL-17A-secreting CD4+ T cells,^63,64^ DQ8-restricted gut microbiota may increase disease severity in HLA-DR3.DQ8 mice through the induction of IL-17A. Thus, HLA class-II may affect MS susceptibility by influencing gut microbiota composition and associated cytokine networks.

In addition to the HLA genes, approximately 200 autosomal non-MHC gene variants have been reported as possible risk alleles for MS,^8,65^ which may modulate gut microbiota composition. However, a direct link between these gene variants and gut microbiota is lacking.

### 4.2. Dietary factors, gut microbiota, and multiple sclerosis

**Figure 2.**
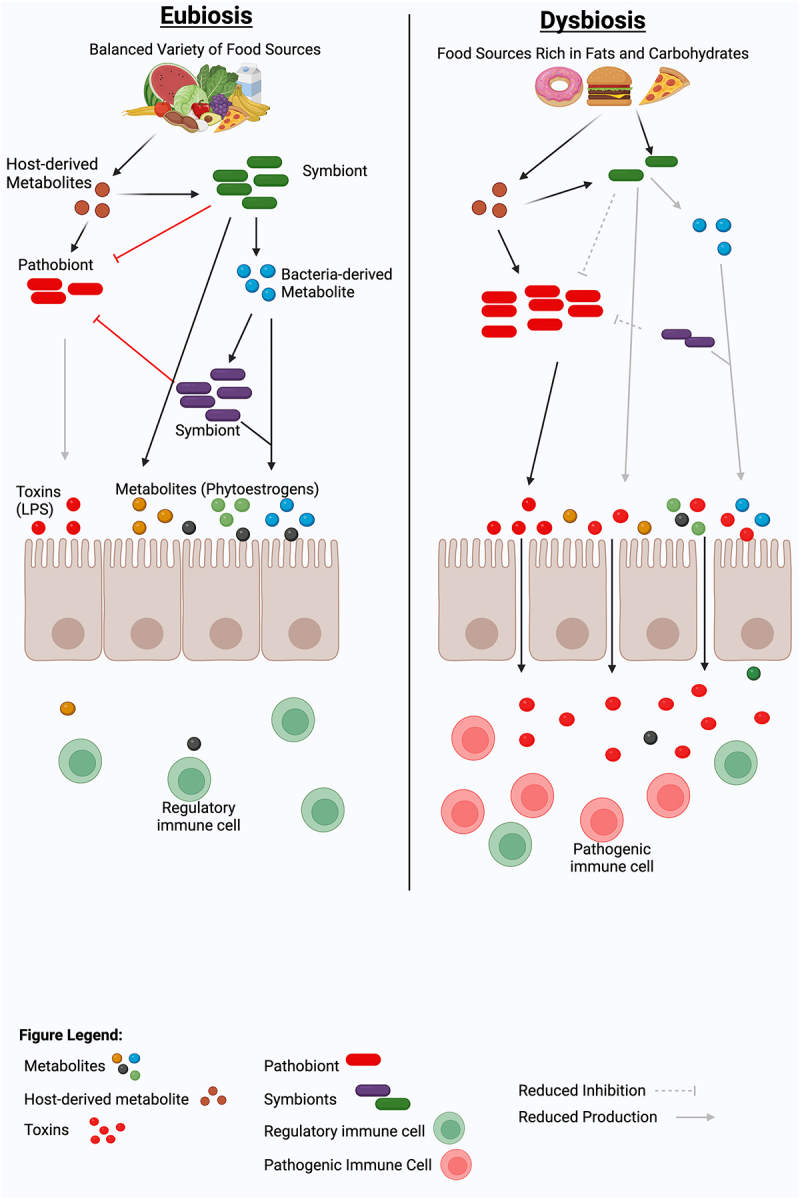
Dietary modulation of gut microbiome and metabolites for immune balance and Eubiosis. A healthy diet, such as a diet rich in fibers, isoflavones, or tryptophan, can promote a diverse, balanced gut microbiota that can maintain a healthy eubiotic state by inducing immunoregulatory cells and cytokines. In contrast, a lack of beneficial plant metabolites in the diet or enrichment of a high-fat or high-fructose diet can induce a dysbiotic gut microbiota characterized by the loss of beneficial gut bacteria and the acquisition of immunostimulatory bacterial molecules such as lipopolysaccharide (LPS). This dysbiotic gut microbiota can predispose or propagate the disease by inducing pathogenic immune cells, which can induce local and/or systemic pro-inflammatory responses. Figure created with BioRender.com.

Among all factors linked with the gut microbiota, the diet has emerged to have the strongest influence on the composition and function of the gut microbiome (Figure 1). As our ancestors consumed diverse plant-based diets, gut microbes co-evolved, providing essential enzymes for digesting complex fibers and unlocking additional food sources.^66,67^ Adapting to varying environments, this symbiotic relationship transformed humans into holobionts, relying on gut bacteria for functions like vitamin production, nutrient digestion, and immune regulation.^68^ Since the gut microbiome is so intertwined with human physiology, any disruption to this delicate balance can have far-reaching consequences. Environmental changes, particularly those related to diet, can disrupt the delicate balance of the gut microbiome (dysbiosis), which has been associated with a growing number of diseases, including MS.^69^ A diet rich in components like fiber, phytoestrogens, tryptophan, fats, and sugars, significantly shapes the composition and function of our gut microbiota (Figure 2), which in turn plays a vital role in our overall health. In this section, we will discuss how these dietary elements can either positively or negatively impact our gut microbiome and, consequently, pathobiology of MS. Additionally, we will discuss how gut bacteria-mediated bile acid metabolism can influence both immune function and neuromodulation (Figure 2).

#### 4.2.1. Fibers

Dietary fiber, the indigestible part of plant foods, is a vital fuel source for the beneficial bacteria residing in our gut.^70,71^ When gut bacteria ferment these complex carbohydrates, they produce SCFAs, including acetate, propionate, and butyrate. SCFAs play a multifaceted role in maintaining human health. They provide energy for our gut cells, help regulate blood sugar levels, boost our immune system, and reduce inflammation.^72^ Additionally, SCFAs influence gut barrier function, protecting against harmful pathogens and potentially signaling to our brains, influencing appetite and mood.^72^

SCFAs are known to provide many beneficial functions to the host both locally and systemically. One of the major functions linked with SCFAs, specifically butyrate and propionate, is their ability to induce Treg generation in the colon and periphery.^73,74^ SCFAs mediate this effect through G protein-coupled receptors and Histone Deacetylase (HDAC) inhibition, leading to decreased TLR signaling and repression of the NLRP3 inflammasome. As a result, SCFAs can reduce excessive inflammatory responses and promote anti-inflammatory responses, including the generation of Tregs.^73^ SCFAs are also critical for intestinal barrier stability, regulating the production of IL-10 and IL-22 by activating Signal Transducer and Activator of Transcription 3 (STAT3) and promoting epithelial regeneration and anti-microbial peptide production.^75^ Overall, SCFAs have potent immunomodulatory activity critical to maintaining gut homeostasis.

SCFAs are mostly absorbed and metabolized by colonocytes, and only a small portion of SCFAs reach systemic circulation. In germ-free (GF) mice, microglia appear stunted, and SCFA supplementation was sufficient to induce their maturation.^76^ Additionally, antibiotic-induced perturbations to gut microbiota have been shown to influence neuroinflammation and affect microglia.^77,78^ In vitro, butyrate treatment has shifted microglia to a more anti-inflammatory profile characterized by reduced IL-1β, IL-6, and TNF-α expression.^79^ SCFAs also regulate the expression of tryptophan 5-hydroxylase, the rate-limiting enzyme in the serotonin production.^80^ Furthermore, SCFAs, especially butyrate, enter the brain via monocarboxylate transporters (MCTs) found on the blood–brain barrier and central nervous system cells. MCT-1, present in both intestinal and CNS cells, especially oligodendrocytes, astrocytes, and neurons,^81^ transports butyrate and lactate, acting as an energy source and supporting oligodendrocyte survival. MCT-2, mainly in neurons, exclusively transports butyrate and is involved in synapse repair.

In MS, both total SCFA production and the SCFA profile are altered and characterized by decreases in either acetate, butyrate, or propionate.^17,82,83^ Strikingly, in one study, propionate supplementation to therapy-naïve PwMS led to a significant increase in functionally competent Tregs and a decrease in Th1 and Th17 cells. Post-hoc analysis also showed that propionate supplementation reduced the annual relapse rate.^17^ Given that glial cells like microglia, astrocytes, and oligodendrocytes are key players in the pathobiology of MS, the ability of SCFAs to influence the function of these cells, along with neurons, suggests a potential neuroprotective role of SCFA in MS.

In summary, SCFAs may modulate MS disease severity in various ways, including neuro and immune modulation to maintain an anti-inflammatory and neuroprotective phenotype. However, further research is needed to elucidate the precise mechanism through which the neuroactive and immunomodulatory capabilities of SCFAs ameliorate clinical disease.

#### 4.2.2. Phytoestrogens

Phytoestrogens are plant-derived polyphenols with structural similarities to human estrogens and are comprised of several classes of chemical compounds, such as isoflavones (soy) and lignans (flaxseed).^84,85^ Humans do not have the capacity to metabolize phytoestrogen, but certain gut bacteria can metabolize them to produce metabolites such as S-equol from isoflavones.^86^ Daidzein and Genistein are the two important isoflavones and are often present as glycosides or aglycones in plant products. These isoflavones are metabolized by gut bacteria belonging to the genera *Adlercreutzia*,^87^ *Bifidobacterium*,^88^ *Eggerthella*,^89,90^ *Lactobacillus*,^91^ *Slackia*,^92^ etc. to end products S-equol and/or O-DMA depending upon the dietary habits and type of gut bacteria present.^93^ Importantly, phytoestrogen metabolizing bacteria *Prevotella, Parabacteroides, Adlercreutzia, Slackia*, and *Lactobacillus* were lower in PwMS compared to healthy controls.^39,42^

Phytoestrogens have shown immunomodulatory and neuroprotective effects in multiple studies.^85,94^ Phytoestrogen and their metabolites can interact with estrogen receptor alpha (ERα) and estrogen receptor beta (ERβ)^95^ that are expressed in human and mouse immunogenic cells, including T cells, B cells, Natural Killer (NK) cells, macrophages, and dendritic cells (DC).^96^ S-equol has a strong estrogenic,^97^ antioxidant,^98^ and anti-androgenic^99^ activity. We have summarized the effect of phytoestrogen compounds on these cells previously,^85^ which indicates that they can exert both local and systemic immunological effects.

While the neuroprotective effects of equol have not been directly studied in MS or EAE, studies in other neuroinflammatory diseases and models suggest its neuroprotective potential.^20,21,100^ S-equol can reduce neuroinflammation through modulation of the TLR4/NF-kappaB pathway, restoration of neurotransmitter balance, and promotion of synaptic plasticity.^20^ Additionally, equol has been shown to mediate neuroprotective properties by reducing neuronal death and promoting neurite outgrowth, possibly by enhancing neurotrophin production in astrocytes.^20^

Phytoestrogens, especially isoflavones, have been shown to exert anti-inflammatory effects in the gut and protect from EAE.^101–104^ We have shown that the disease-protective effect of isoflavones was dependent on the presence of gut bacteria, especially those with the ability to metabolize dietary isoflavones into S-equol.^104^ Furthermore, we have shown that dietary isoflavones can modulate the gut microbiota to enrich beneficial bacteria and reduce EAE severity by altering lipopolysaccharides (LPS) biosynthesis.^103^ In addition, dietary isoflavones have been shown to reduce inflammation by modulating phenylalanine and lipid metabolism.^105^ Presently, there is a lack of research investigating the connection between phytoestrogen levels in PwMS and disease severity, as well as the potential benefits of phytoestrogen supplementation in MS. Future studies may shed light on the importance of phytoestrogens and their metabolism by gut bacteria in the context of MS.

In conclusion, the complex interplay between dietary phytoestrogens, gut microbiota, and their metabolites highlights the potential for significant influence on immune regulation and neuroprotection. The observed reduction of phytoestrogen-metabolizing bacteria in PwMS suggests a potential link between gut dysbiosis and an impaired ability to generate beneficial metabolites like S-equol. While S-equol’s immunomodulatory effects are of interest, the recent findings about isoflavone-induced alterations in LPS biosynthesis underscore the multifaceted mechanisms through which the gut microbiome mediates the beneficial effects of phytoestrogens. Further research is crucial to fully elucidate these mechanisms, potentially opening doors to novel therapeutic approaches for PwMS, targeting both the gut microbiome and phytoestrogen-derived metabolites.

#### 4.2.3. Tryptophan

L-tryptophan is an essential amino acid found both in meat and plant-based foods and is crucial for various physiological functions.^106^ It can be absorbed from the gut into the bloodstream, and once in circulation, it is transformed through various metabolic pathways, including the kynurenine and serotonin pathways, contributing to numerous physiological processes.^22^ Within the gut, tryptophan serves as a substrate for metabolism by resident bacteria, leading to the production of beneficial metabolites.^106^ Gut microbiota catabolizes tryptophan into tryptamine and various immunomodulatory indole derivatives, including indole-3-aldehyde, indole-3-acetic-acid, and indole-3-propionic acid.^107^ These indole derivatives exert their functions by activating the aryl hydrocarbon receptor (AhR), leading to many downstream events essential for gut homeostasis.^108^ *Lactobacillus* species, bacteria known to catabolize tryptophan, were shown to attenuate gut inflammation via AhR and regulate IL-22 production to protect against fungal infection.^109,110^ Additionally, *Lactobacillus reuteri* were found to reprogram intraepithelial CD4+ T cells into immunoregulatory cells via indole derivatives.^111^ Tryptophan catabolites have also been shown to regulate intestinal barrier integrity. Specifically, IPA was found to protect barrier function in a mouse model of colitis via the pregnane X receptor and reduce intestinal permeability in mice fed a high-fat diet.^112,113^

Additionally, gut microbiota can also influence serotonin synthesis, a tryptophan derivative, where SCFA production increases the expression of Tryptophan Hydroxylase 1 (TPH1), leading to increased serotonin levels.^80,114^ Certain gut bacteria have been shown to produce serotonin, including *Lactobacillus*, *Lactococcus*, and *Streptococcus* species.^115^ Importantly, serotonin can attenuate EAE severity by reducing IFNγ production and T cell proliferation.^116,117^

Tryptophan can also directly affect the CNS resident cells through the kynurenic acid pathway.^22^ While in a homeostatic condition, kynurenic acid is neuroprotective; during inflammation, kynurenine, a precursor to neurotoxic metabolites like quinolinic acid, is produced by microglia and macrophages in the CNS. Additionally, the tryptophan-derived indole-containing metabolites can induce inflammatory pathways in microglia and astrocytes through the aryl hydrocarbon receptor (AhR).^22^

In MS, it has been shown that PwMS have lower urinary levels of kynurenine, a known immunosuppressive tryptophan metabolite, which was also negatively correlated with the Expanded Disability Status Scale (EDSS) score.^118^ In the CNS, Quintana et al. found that supplementation with tryptophan metabolites activated AhR on astrocytes leading to an increase in IFN-I signaling and attenuating EAE.^119^ Importantly, AhR agonists, including tryptophan metabolites were also reduced in PwMS.^119^ Interestingly, high tryptophan diet ameliorated EAE and reduced autoreactive T cell activation and migration. Although, these effects were independent of the AhR.^120^ However, the protective role of AhR activation in CNS autoimmunity is still controversial as *Lactobacillus reuteri* supplementation can enhance IL-17 production from CD4+ T cells and exacerbate EAE through Ahr activation.^121^

Altogether, tryptophan catabolism by the gut microbiota is known to have both local and systemic immunomodulatory as well as neuroprotective effects, with the potential to modulate MS disease severity. However, further research is necessary to understand the significance of host and microbial tryptophan metabolism in MS.

#### 4.2.4. High-fat and high-fructose diet

A high-fat diet (HFD), characterized by an excessive intake of fats, particularly unhealthy saturated and trans fats plays a significant role in the rising global incidence of obesity (affecting 650 million people worldwide, with 100 million in the USA) and contributes to inflammatory diseases, including MS.^122,123^ Obesity has evolved into a global public health crisis, with approximately 35% of adults in the U.S. classified as obese, as reported by the Centers for Disease Control and Prevention (CDC) in 2023. Numerous studies over the last decade have also elucidated the significant impact of obesity as a risk factor for both the susceptibility and severity of MS.^124–126^

HFD can significantly impact gut barrier integrity and mucosal immune responses.^127–129^ HFDs have been shown to disrupt the tight junctions between intestinal epithelial cells, leading to increased intestinal permeability, often referred to as “leaky gut”.^128^ This increased permeability allows for the translocation of harmful substances, such as bacterial toxins (e.g., lipopolysaccharides) and metabolites, from the gut lumen into the bloodstream, triggering systemic inflammation. Furthermore, HFDs can alter the composition of the gut microbiota, promoting the growth of pro-inflammatory bacteria and suppressing beneficial species.^128,129^ Our recent study on HFD-induced obesity in mice showed gut dysbiosis with enrichment of *Proteobacteria* and *Desulfovibrionaceae* and reduced abundance of crucial bactiera that maintain a healthy gut milieu, such as *Lactobacillus, Prevotellaceae*, and *Muribaculaceae*.^129^ This dysbiosis can further exacerbate mucosal immune responses, leading to chronic low-grade inflammation, which is implicated in autoimmune diseases including MS. Notably, the depletion of dysbiotic gut microbiota in HFD-fed mice ameliorated disease severity, underscoring the pivotal role of gut dysbiosis in exacerbating MS susceptibility and severity in the context of HFD-induced obesity.^129^ HFD induced obesity in mice has been linked to enhanced microglial activation and an increase in pro-inflammatory Th1 and Th17 cells, key players in EAE.^127^ Furthermore, obesity has been shown to promote EAE through increased levels of IL-6 and CCL-2, cytokines that facilitate the infiltration of T cells into the central nervous system, exacerbating inflammation.^127^ HFD have been linked to disruption of the blood–brain barrier (BBB) and increased oxidative stress, both of which are implicated in neurodegenerative diseases.^130–132^ There are limited studies on the ability of HFD-induced obesity to modulate glial cells and neurons in MS or its animal model. However, based on studies in other models of neurological diseases where HFD-induced obesity has been shown to modulate microglia, astrocytes, oligodendrocytes, and neurons.^130–132^ This suggests that similar pathways might be activated in MS and EAE. In the context of EAE and MS, a compromised BBB could allow for increased infiltration of immune cells and inflammatory molecules into the central nervous system, exacerbating neuroinflammation.

Besides high fats, a diet rich in sugar, especially high fructose syrup has also emerged as an important factor in obesity.^133^ Although, there are no studies on the role of sugar intake on MS, a study in patient with neuromyelitis optica spectrum disorder has shown a link between higher sugar intake and disease severity.^134^ We have analyzed effect of Fructose-rich diet (FRD) in mice and EAE model.^135^ We have shown that mice on FRD lost beneficial bacteria such as *Prevotella, Muribaculum*, and *Bifidobacterium*^135^ and enriched for *Desulfovibrio, Collinsella, Olsenella*, and *Bacteroides* species. Additionally, mice on FRD show enrichment of immune populations linked with pro-inflammatory phenotypes such as Helios-RORγt+FoxP3+CD4+ T cells in the small intestine.^135^ Surprisingly, despite the changes observed in the gut microbiota and immune system, an FRD had only a minor impact on the severity of EAE. Thus, further study is warranted to determine the precise role of high fructose on the pathobiology of EAE and MS.

In conclusion, the gut microbiome plays a crucial role in how diets high in fat or fructose negatively impact health. Interestingly, certain bacterial taxa, such as *Desulfovibrio*, *Prevotellaceae*, and *Muribaculaceae*, were altered in both the HFD and FRD groups, indicating a possible shared pathway for triggering inflammatory responses. A better understanding of how these diets alter the composition and function of gut bacteria could pave the way for improved treatments for obese PwMS.

### 4.3. Bile acids

Bile acids (BA) are amphipathic molecules generated in the liver and stored in the gall bladder after cholesterol breakdown.^136^ There are two mechanisms for BA synthesis. The classical pathway produces primary BA, which conjugates with glycine and taurine in the liver before being stored in the gall bladder.^137,138^ BA is released from the gall bladder and into the small intestine, which aids digestion and absorption of nutrients.^137^ If BAs are not absorbed in the intestines, the alternative pathway occurs where gut microbiota converts BAs into secondary BAs. Eventually, the secondary BAs will be absorbed in the colon and later return to the liver for enterohepatic circulation.^139^

BA composition and levels are important for regulating gut diversity and homeostasis.^138^ The gut microbiome can deconjugate, dehydroxylate, and dehydrogenate BAs.^139^ Bacteria, such as *Lactobacillus*. spp., *Bifidobacterium* spp., and *Enterococcus* spp., deconjugate BA (remove glycine and taurine) in the small intestine via bile salt hydrolases.^140,141^ After deconjugation, some bacteria, like *Clostridium* spp., can convert cholic acid and chenodeoxycholic acid (CDCA) into deoxycholic acid (DCA) and lithocholic acid (LCA) via dihydroxylation.^138^ Gut microbes will dehydrogenate and epimerize CDCA to ursodeoxycholic acid while also converting DCA and LCA to iso-DCA and isoLCA.^142,143^ These processes increase BA solubility, which is necessary for BA functions.^144^ BAs have broad functions, including interacting with cell surface and nuclear receptors, membranes, the immune system, the nervous system, and the gut microbiome.^145,146^ Additionally, BA composition can be affected by diet, sex differences, and antibiotic treatment. High- and low-fat diets can decrease primary BA synthesis, while high-protein diets can increase DCA, LCA, and CDCA levels/BA synthesis.^147,148^ BA composition also varies with age and sex. Decreased BA levels are commonly found in men but not women.^149^

In recent studies by Bhargava et al., human BA levels were reported to be abnormal in PwMS, and their receptors are present in MS brain lesions.^136^ In PwMS, gut dysbiosis was associated with the presence or absence of BA-metabolizing bacteria.^38,150^ PwMS harbor different gut microbiota depending on their stage of disease. Specifically, Chen et al. found BA-metabolizing bacteria, like *Parabacteroides* and *Erysipelotrichaceae*, to be decreased in PwMS.^39,42^ The absence of these bacteria can result in decreased BA metabolism and metabolite production necessary to support homeostasis at mucosal surfaces.^151^ In EAE, the animal model of MS, neuroinflammation is associated with altered cholesterol metabolism in astrocytes and abnormal circulating BA metabolites.^152^

In contrast to conjugated primary BA, gut bacteria-derived unconjugated bile acids readily enter the CNS via a simple diffusion.^22^ Different bile acids have distinct effects on BBB permeability, with unconjugated CDCA and DCA increasing permeability, while both unconjugated and glycine-conjugated ursodeoxycholic acid (UDCA) strengthen the BBB. Recent studies suggest that BA supplementation, particularly with tauroursodeoxycholic acid (TUDCA), can reduce neuroinflammation.^136^ TUDCA supplementation ameliorated EAE and limited pathogenic inflammatory pathways in glial cells through G protein-coupled bile acid receptor 1 (GPBAR1) signaling.^136^

In conclusion, gut microbiota-derived bile acids metabolites represent a complex and multifaceted class of molecules with significant roles in neurological diseases like MS. The observed dysbiosis in PwMS, particularly the changes in bile acid-metabolizing bacteria, suggests a crucial interplay between the gut microbiome, bile acid profiles, and neuroinflammation.

## 5. Mechanisms through which gut dysbiosis promotes inflammation and disease

During a healthy state, the gut is lined with intestinal epithelial cells (IECs) that separate microbes and their products from host cells/tissues due to tight junctional proteins.^153^ However, dysbiosis may promote a cascade of events, including the enrichment of pathogenic bacteria and the release of deleterious toxins, leading to a pro-inflammatory environment and a compromised gut barrier.^154,155^ A key player in this process is thought to be pathogen-associated molecular patterns (PAMPs), including LPS, a component of gram-negative bacteria.^156^ This increased gut permeability (Leaky gut) permits PAMPs such as LPS and other bacterial metabolites to enter the bloodstream, triggering widespread systemic inflammation. Additionally, certain bacteria can directly influence the immune system, shaping the development and behavior of immune cells such as CD4 T cells, B cells, dendritic cells (DCs), and macrophages.^157^ The combination of LPS-induced inflammation, leaky gut, and immune activation creates a perfect storm for dysregulated immune activation that can fuel chronic diseases. Furthermore, dysbiosis can lead to changes in the metabolites produced by gut bacteria, and a reduction in health-promoting metabolites, like short-chain fatty acids, can contribute to a pro-inflammatory environment.^158^ This chronic state of inflammation sets the stage for the development of various diseases, including MS. Particularly in the context of MS, bacterial metabolites either can help in overcoming tolerance to autoimmune response and/or can contribute to the propagation of disease as discussed below.

### 5.1. Lipopolysaccharides

Lipopolysaccharides (LPS) are surface glycolipids found in the outer membrane of most Gram-negative bacteria. Lipid A, the core oligosaccharide, and the O-antigen comprise LPS. LPS is known to stimulate the immune system and produce a strong immune response mediated mostly through Toll-Like Receptors (TLR)-2 and TLR-4 receptors.^159,160^ The overall structure of LPS is conserved; however, several variations can be found between bacteria in all three structural moieties.^161–163^ Thus, differences in the bacterial populations in the gut microbiota can directly affect the composition and amount of LPS in the gut, determining the local and systemic immune response. Most proinflammatory LPS are derived from *Proteobacteria*, which increase oxidative stress and result in the production of pro-inflammatory cytokines. The binding of LPS to the LPS-binding protein (an acute phase protein) allows the complex to interact with CD14. This complex of LPS-LPS binding protein and CD14 interacts with TLR4/Myeloid differentiation factor 2 (MD-2) at the cell surface, which subsequently activates the cell through the NF-kB signaling pathway, resulting in the secretion of proinflammatory cytokines such as IFN-y, TNF-α, IL-1β, and IL-8.^164^ LPS can directly damage the epithelial barrier locally by inducing the expression of inflammatory cytokines like IL-8.^165^ However, microbiome LPS immunogenicity varies in humans^166^ and such differential immunological responses can be the result of structural differences of LPS.^167^

The pro- and anti-inflammatory nature of LPS has been described in multiple inflammatory conditions, including autoimmune diseases. LPS isolated from gut bacteria, especially *Bacteroides vulgatus* has shown to induce pro-inflammatory endotoxin tolerance acting through MD-2/TLR4 receptor complex in CD11c+ cells of intestinal lamina propria.^168^ Especially in autoimmune diseases, a human study of 222 infants in Northern Europe has shown that LPS isolated from *Bacteroides*, and *Escherichia coli* are structurally different and contribute to the development of autoimmune diabetes differently in mice.^166^ In MS cases, LPS and LPS binding proteins were observed to be higher in the blood.^169^ The same study found these two to be higher in the brain, spinal cord, and blood of rats. Mice exposed to LPS at an early age have also been shown to reduce the severity of MOG-induced EAE at 12 weeks, where authors observed increased IL-10 and FOXP3 transcript levels in the spinal cord.^170^ In addition, we have shown that the isoflavone diet can modulate the composition of gut microbiota and the immunogenicity of LPS.^103^ Specifically, fecal LPS extracts isolated from the gut microbiota of mice fed with an isoflavone diet-induced anti-inflammatory effects by enhancing IL-10 and reducing IL-12/23 in the EAE.^103^ In contrast, fecal LPS from mice on a phytoestrogen-free diet-induced proinflammatory cytokines such as IL-1β, IL-6, TNF-α and IL-12.^103^

Taken together, gut dysbiosis can alter the microbial composition, resulting in either pro- or anti-inflammatory immunological effects based on differences in LPS immunogenicity, consequently affecting the exacerbation or prevention of MS/EAE.

### 5.2. Leaky gut syndrome

Gut dysbiosis can disrupt the homeostasis at mucosal surfaces due to changes in the composition of the gut microbiota.^154^ Specifically, when pathobionts are enriched, and symbionts are depleted, gut barrier dysfunction, or “leaky” gut syndrome (LGS), occurs.^155^ LGS can further be characterized by increased intestinal permeability, allowing for bacterial translocation to occur and the growth and colonization of pathogenic bacteria (pathobionts). When the gut barrier is disrupted, hosts are predisposed to gut-specific as well as systemic and organ-specific diseases including MS.^10^

LGS is commonly characterized by gut-specific inflammation that can manifest diseases like Crohn’s, celiac disease, and colitis.^155^ However, LGS is also observed in patients with neurological diseases such as schizophrenia and autism.^171,172^ Although not completely understood, it is hypothesized that gut dysbiosis can predispose patients to neurological diseases like MS.^173–176^ Besides gut dysbiosis, PwMS have high levels of pro-inflammatory cytokines, like IL-1β, TNF-α, and IL-6, in their sera and increased gut permeability.^177–179^ It is further hypothesized that gut dysbiosis can enhance the activation of the immune system to cause more severe demyelination in the CNS.^173^ Our group and others complement these findings in mice as mice with EAE disease were accompanied by increased gut permeability.^180,181^ Thus, LGS is linked to MS and can play an important role in the pathobiology of disease.

### 5.3. Modulation of regulatory T cell by microbiota

Regulatory T cells (Tregs) play a critical role in suppressing effector T cell responses to self-antigens, thus preventing spontaneous autoimmune diseases. Notably, disturbances in the function of CD4+ Tregs have been observed in PwMS, with several studies reporting a significant decrease in suppressive ability.^182,183^ Additionally, CD8+ Treg function is deficient during acute exacerbation of MS.^16,184^ Thus, increasing Tregs in PwMS or improving Treg functionality remains an enticing potential therapeutic.

Tregs consist of two distinct populations. The first population develops in the thymus, where thymus-derived Treg (tTreg) cells are generated following recognition of self-antigen by the T cell receptor. The second population, peripherally derived Tregs (pTreg), are stimulated in the periphery, where under certain conditions naïve CD4^+^ T cells gain expression of FOXP3 upon recognition of their cognate antigen.^185^ Importantly, pTregs are thought to be dependent on microbiota for expansion and maintenance, whereas gut microbiota composition can dictate pTreg number and function.^185^ Thus, the Treg pool, specifically pTregs, represents a dynamic population that environmental factors may externally modulate.

Evidence for gut microbiota-dependent modulation of Tregs is highlighted by seminal studies showing colonic Treg induction by SCFA-producing bacteria.^186^ SCFA supplementation also increased Treg differentiation and ameliorated EAE, while long-chain fatty acids (LCFAs) exerted the opposite effect.^187^ Additionally, microbiota-induced Treg expansion has been observed in the periphery. Kasper et al. showed that gut commensal *Bacteroides fragilis* enhanced Treg numbers in cervical lymph nodes and, importantly, ameliorated EAE.^188^ Dietary intervention resulting in altered gut microbiota composition has also been shown to modulate Tregs and ameliorate CNS autoimmunity. Piccio et al. observed that intermittent fasting ameliorated EAE and increased Treg frequency in gut-associated lymphoid tissue.^189^ Alterations in gut microbiota composition were highlighted by enrichment of *Bacteroidaceae*, *Lactobacillaceae*, and *Prevotellaceae* families.^189^ However, changes in gut microbiota composition may also indirectly affect the ability of Tregs to restrain autoimmunity through the induction of Th1 and Th17 cells or through modulation of the T cell microenvironment.^190^ For example, we have previously shown that an isoflavone-free diet exacerbates EAE and increases the number of IFNγ and IL-17-producing cells in the CNS. However, Treg frequency and number were not altered.^104^ Further research on the role of microbially induced or expanded Tregs in autoimmunity, as well as a more detailed characterization of Tregs in autoimmune patients, will be necessary to evaluate the ability of microbiome-based therapeutics to modulate the Treg compartment in PwMS.

### 5.4. Pro-inflammatory T cell induction by microbiota

MS is thought to be mediated by self-reactive, myelin-specific CD4+ T helper cells, with Th17 cells being the most implicated lineage.^191^ Th17 cells are characterized by their production of the pro-inflammatory cytokine IL-17 and migrate to the CNS during active disease.^192,193^ However, canonically, Th17s protect mucosal barriers and maintain tolerance toward commensal bacterial flora, making them crucial for intestinal homeostasis.^63^ Thus, it is hypothesized that dysregulation of Th17 differentiation and expansion of Th17 cells, mediated by gut microbiota, may be involved in the initiation or progression of disease.^194^ In support of this hypothesis, germ-free mice are resistant to EAE and lack Th17 cells.^18^ However, mono-colonization with segmented filamentous bacteria (SFB) was sufficient to restore susceptibility to EAE disease and led to the expansion of Th17s in the CNS.^18^ These data describe a requirement for gut bacteria in EAE pathology and suggest that immunostimulatory bacteria that expand Th17 cell populations may influence clinical disease. Furthermore, Duc. et al. have shown that disrupting myelin-specific Th17 cell trafficking to the colon during EAE significantly attenuates disease, suggesting a role of gut microbiota in catalyzing the encephalitogenic properties of Th17 cells.^195^ Interestingly, bacterial species that can promote Th17 induction and pro-inflammatory processes have been associated with MS, including *Akkermansia muciniphila* and *Acinetobacter calcoaceticus.^38^ Akkermansia muciniphila* colonization has also exacerbated EAE *in vivo*.^104^ These immunostimulatory bacteria may directly induce Th17 cell responses or indirectly through metabolite production. In one study, *Lactobacillus reuteri* tryptophan metabolism promoted CNS autoimmunity through aryl hydrocarbon receptor (AhR) stimulation leading to increased IL-17 production.^121^ Understanding how gut bacteria exacerbate clinical disease through the induction of pathogenic Th17 cell populations and the amplification of pro-inflammatory cytokine production or through the expansion of autoreactive Th17s will be critical.

### 5.5. Regulatory and pro-inflammatory B cells induction by microbiota

B cells have emerged as an important immune cell in the pathogenesis of MS. Specifically, abnormalities have been observed in the quantity and quality of immunoglobulins in the cerebrospinal fluid (CSF) where greater than 90% of patients with MS are positive for Immunoglobulin G (IgG) oligoclonal bands in the CSF.^196^ B cells also represent a subset of infiltrating cells in the brain and spinal cord of PwMS.^197^ The significance of these abnormalities is emphasized by the efficacy of B cell depletion therapies in PwMS and the compelling evidence linking Epstein–Barr virus (EBV) infection and anti-EBNA antibody levels to the onset of clinical symptoms.^198,199^ However, the role of gut microbiota on pathogenic B cell responses in EBV or MS is poorly understood and requires further study.

A gut microbiota-dependent, anti-inflammatory role of B cells in MS has been elucidated. Rojas et al. found that IgA+ plasma cells (PCs) are significantly reduced in the gut during EAE and, importantly, the removal of plasmablasts and PCs resulted in exacerbated EAE.^200^ A follow-up study found that IgA+ B cells traffic across the blood–brain barrier during active MS and have specificity toward MS-associated immunostimulatory bacterial strains. However, these IgA+ B cells were potent IL-10 producers and do not cross-react with the self-antigen.^201^ Interestingly, IgA production also appears to be altered in the gut of PwMS, where an increased EDSS score is associated with a decrease in gut IgA-coated bacteria.^202^

These data highlight the complex role B cells play in the pathobiology of MS. Microbially induced IgA+ B cells may represent a regulatory subset in the CNS, and B cells of other specificities, such as EBV-specific B cells, may exacerbate disease through chronic inflammatory cytokine production and self-antigen cross-reactivity.

## 6. Microbiome based therapeutics

The gut microbiome is known to influence the pathogenesis of many human diseases, and there is significant evidence supporting gut microbiota alterations in PwMS.^10^ Thus, targeted interventions to modulate gut microbiota toward a “healthier” composition remain an attractive potential therapy. A variety of approaches are used to modulate gut microbiota, including diet, probiotics, synbiotics, and fecal microbiome transplantation (FMT), all with potential in MS. Multiple dietary regiments have been explored in MS to modify gut microbiome composition and downregulate systemic inflammation.^203^ The Swank diet, a low-fat diet developed by Dr R. L. Swank, restricts fat consumption to less than 20 g per day in an attempt to reduce cholesterol and improve cerebrovascular health. Importantly, Dr Swank performed a 34-year follow-up study showing that PwMS who consumed less than 20 g of fat were less likely to experience severe exacerbations or death.^204^ However, Swanks’ studies were not randomized controlled trials and may be biased for a multitude of reasons. The Wahls diet, a modified paleolithic diet developed by Dr Terry Wahls, has also been shown to improve MS symptoms. In a small, randomized waitlist-controlled trial, PwMS exhibited improved fatigue and QOL scores after 3 months on the diet.^205^ The diet also has the potential to exacerbate the disease. For example, obesity significantly increases the risk and severity of MS, and a high-fat diet, linked with increased incidence of obesity, was shown to induce gut dysbiosis and exacerbate EAE in mice.^39,129^ However, further study is necessary to understand how diet quality impacts the development and severity of MS symptoms. Probiotic-based therapies have also been used to treat PwMS, and probiotics can ameliorate EAE via multiple mechanisms.^117,180^ Interestingly, *Prevotella histicola* supplementation was shown to be as potent as COPAXONE in ameliorating EAE and reduced demyelination and inflammation in the brain.^206^ However, clinical data on the beneficial effect of probiotics in PwMS has been inconclusive. A meta-analysis of three randomized controlled trials was performed to demonstrate differences between PwMS receiving probiotic or placebo supplementation. While improvements were seen in levels of inflammatory and oxidative stress markers, differences in EDSS and Beck Depression Inventory (BDI) scores were heterogeneous.^207^

However, in another meta-analysis, probiotics were shown to significantly improve depression and anxiety in PwMS.^208^ More randomized controlled trials and better approaches to identify immunomodulatory bacteria in PwMS with the potential to modulate disease are necessary. A combination of dietary alterations and probiotic supplementation may also be important to promote the growth of beneficial gut bacteria, commonly known as synbiotic therapy. This approach has shown promise in animal models of MS where *Parabacteroides distasonis* and *Aldercrutzia equolifaciens* supplementation in conjunction with an isoflavone-rich diet ameliorated EAE. In contrast, *Escherichia coli* supplementation and isoflavone diet did not ameliorate EAE.^104^ FMT is another exciting potential therapy that has shown promise in GI diseases such as *Clostridium difficile* infection. However, defining what constitutes a “healthy” microbiome with the potential to suppress inflammation and MS remains a challenge.

## 7. Conclusions

The significance of gut microbiota in the pathobiology of MS is increasingly recognized, presenting a vast potential for leveraging its capabilities as a potential diagnostic, prognostic, and therapeutic tool. However, further investigation is warranted to delineate specific bacteria or bacterial communities associated with the disease, both positively and negatively, as this understanding will be pivotal in harnessing their potential effectively. While the gut microbiome has garnered significant attention in the context of MS, it is crucial to acknowledge that other host microbiomes, such as the oral^209–211^ and nasal microbiota,^211^ may also play a role in the development and progression of this neurological disorder. Emerging research suggests that alterations in the composition and function of these microbial communities could influence immune responses and potentially contribute to neuroinflammation.^212^ Investigating the interplay between these diverse microbiomes and the host immune system represents a promising avenue for future research, potentially uncovering novel insights into MS pathogenesis and paving the way for innovative therapeutic interventions targeting these microbial ecosystems. Additionally, prospective investigations into elucidating the interplay among various gut bacteria affected in PwMS will aid in delineating a disease-modifying gut microbiome. Such studies will also contribute to determining whether a combination of diet and microbiota (synbiotics) may present a more effective treatment strategy for PwMS compared to solely relying on bacteria (probiotics) or diet (prebiotics).
